# Using the Candidacy Framework to Explore Access to NHS Healthcare for Street Sex Workers in Sheffield: An Ethnography and Art-Based Research Project

**DOI:** 10.3390/healthcare14030387

**Published:** 2026-02-03

**Authors:** Camille Ball, Rebecca L. Mawson, Josephine Reynolds, Louise Millington, Beth Webster

**Affiliations:** 1 Primary Care Research, Division of Population Health, School of Medicine and Population Health, University of Sheffield, Regent Court, Sheffield S1 4DA, UK; 2 Forge Health Group, 151 Burngreave Rd., Sheffield S3 9DL, UK; 3 Sheffield Working Women’s Opportunity Project, WMS House, 61A Wicker, Sheffield S3 8HT, UK

**Keywords:** healthcare access, health inequalities, stigma, marginalised populations, ethnography, primary care

## Abstract

**Highlights:**

**What are the main findings?**

- Rather than being disengaged from healthcare, street sex workers face systematic barriers across all stages of healthcare access. This includes minimisation of health needs, administrative exclusion through digital systems, lack of consistent care, and stigma from professionals.
- Key enablers of healthcare access include supportive organisational staff, trusted relationships with consistent female healthcare providers, and trauma-informed care delivered through accessible third-sector partnerships.

**What are the implications of the main findings?**

- Healthcare services must be redesigned through co-production with street sex workers. This includes the need to simplify registration processes, provide in-person and non-digital options, and prioritise continuity of care over utilisation metrics when evaluating service effectiveness.
- Service provision should recognise that street sex workers are systematically excluded rather than disengaged. Interventions require trauma-informed, gender-sensitive approaches with flexible appointment systems and training for healthcare professionals on the specific triggers and needs of this marginalised population.
- Specific trauma-informed communication skill sessions should be delivered to reduce the potential for stigmatising or triggering communication.

**Abstract:**

**Background**: Street sex workers (SSWs) experience some of the highest levels of health inequality in the UK, yet face persistent barriers to accessing NHS healthcare. These barriers are shaped by structural disadvantage, stigma, and the complex realities of their lives. Despite significant health needs, engagement with services remains low, and existing models of care often fail to accommodate the lived experiences of this population. **Aims**: This study explores how SSWs access, experience, and navigate NHS healthcare. It aims to understand the barriers and enablers of access, identify areas for improvement, and offer recommendations to inform the development of more inclusive service provision. **Methods**: An ethnographic approach was undertaken within a South Yorkshire charitable organisation. Data collection involved participant observation and an arts-based scrapbook intended to facilitate trauma-informed, flexible engagement. Thematic analysis was used to analyse the data, organised around a dynamic, processual approach using the candidacy framework. **Findings**: Barriers to care were present across all stages of healthcare engagement, including minimisation of health needs, administrative exclusion, lack of continuity, and stigma from professionals. Participants frequently described systems as inaccessible. Key enablers included supportive organisational staff and consistent, trusted relationships with specific providers. **Areas for Improvement and Recommendations**: Findings highlight the need to simplify registration processes, provide in-person options, and reduce reliance on digital communication. Greater continuity of care and gender-sensitive, trauma-informed approaches were consistently requested. Services should not be evaluated solely by uptake but by how well they accommodate marginalised users. Healthcare settings that prioritise safety, trust, and consistency were shown to improve engagement. SWs spoke of the work of accessing care, which for many was too hard to gain. **Conclusions**: SSWs are not disengaged from healthcare but are routinely excluded by systems that fail to meet their needs. Service redesign must begin from the realities of those who are most marginalised, through co-production, to reduce health inequity and build meaningful access.

## 1. Introduction

Sex work (SW) is a broad term for the exchange of sexual services for financial (or equivalent) return. Equivalent payments may include, but are not limited to, shelter, security from others, drugs/alcohol or food [1]. Sex work encompasses a large variety of work types and associated experiences (Table 1) [1]. In the UK, around 95% of SWs are cis or trans women, and we focus on this group in our paper [2].

SWs are a highly marginalised group [3]. The likelihood of becoming involved with SW, particularly forms of SW with greater risk like street work, increases with disadvantage [4]. Multiple disadvantage is prevalent in this community, and they often have a history of unstable or volatile home environments, homelessness, poverty, violence and trauma [5]. Trafficking for sexual exploitation (the movement of someone to another place for the purposes of exploitation) is estimated to affect 13% of females in the UK sex industry [2]. Street sex workers (SSWs) often face greater vulnerability than other SW groups, shaped by distinct experiences, with many facing early-life trauma, with high rates of domestic and sexual abuse [4,6]. Entry into SSW frequently reflects these unstable beginnings, with an average starting age of 19 [4]. SSWs are at extremely high risk of abuse from clients—they report putting their lives at risk for every interaction [6]. Violent experiences have been documented, including being tied up, beaten, strangled, raped and attacked with weapons [4]. This allows for a cycle of women with multiple disadvantage being vulnerable to further exclusion, which can marginalise them further [5].

### 1.1. Health Needs of Street Sex Workers

SSWs are vulnerable to multiple health risks, yet their engagement with healthcare is low [7,8,9]. Unscheduled and emergency care attendance and admission rates are higher amongst SWs than the general population [7]. Presentations tend to be delayed, leading to more severe and irreversible pathology [10]. The prevalence of psychiatric health issues, including depression, post-traumatic stress, anxiety and suicidality, is higher in SWs than the general population [11]. It is noted that pre-existing mental disorders are also associated with entry into SW [12,13].

SWs are more likely to use drugs and engage in high-risk drug behaviours such as intravenous use compared to the general public [14]. A barrier to mental health services for SSWs is the frequency of dual diagnosis—coexisting mental illness and substance misuse—which often excludes them from mental health services until substance misuse is addressed [3]. However, many rely on substances to self-manage existing mental health conditions [15]. Oftentimes, drugs and alcohol will be used to cope with and forget about the traumatic nature of the work [16]. The use of drugs and alcohol as a coping mechanism can cause SSWs to become trapped in the ‘work-score-use’ cycle [4], perpetuating drug addiction and unsafe behaviours.

The sexual and reproductive health needs of SSWs are unique in that they are directly linked to occupational risk. Sexually transmitted infection (STI) is prevalent due to high amounts of unprotected intercourse [17,18]. The prevalence of lifetime pregnancy is high, as are abortion rates [19]. SWs are more likely to have complications during pregnancy [8]. Contraception use amongst SWs is complex. Whilst uptake can be low [18], many SWs are highly aware of STI and pregnancy risk [20]. Menstrual periods are also a consideration within contraception choice, as they impact occupationally [20].

### 1.2. The Candidacy Framework

Dixon-Woods defines candidacy as ‘the ways in which people’s eligibility for medical attention and intervention is jointly negotiated between individuals and health services’ [21]. The candidacy framework helps us explore the varying stages of a patient’s healthcare journey, factoring in limitations to healthcare access [21]. The framework consists of seven stages, which are dynamic and can overlap, reflective of the access process [22]. The stages of the candidacy framework are outlined in Table 2. We have experience of applying this framework to conceptualise access to sexual and reproductive health in primary care [23], and this study fits well with the theoretical components of the framework [23].

## 2. Materials and Methods

### 2.1. Study Setting

This study was a mixed-methods qualitative study, which combined an ethnographic approach and art-based research (ABR). Participants were all users of Sheffield Working Women’s Opportunity Project (SWWOP), a third-sector organisation that offers support for SSWs in Yorkshire. SWWOP interacts with approximately 150 women across two sites: a drop-in centre and an outreach van. In 2025, the age range of women accessing services is between 19 and 71. The charity has dedicated housing and substance use support workers, a dedicated GP and outreach from sexual health to provide STI testing and condoms. They also provide women with essentials such as food parcels, clean/dry clothes, toiletries and menstrual products.

We worked closely with charity members, and organisational consent was obtained prior to beginning data collection. The organisation hosts a GP (LM) to provide primary care for women attending; this is funded by short-term contracts with the local authority and local funding streams. Our research team has worked extensively with underserved populations and has recently presented a critical analysis addressing the power imbalance in research, which was used to devise this project [24,25]. We designed the project based on the needs of the population rather than those of the academic institution, which enabled women traditionally excluded from research to share their voices. Our article authorship reflects that model.

During the six-month ethnographic fieldwork period (October 2024–April 2025), CB engaged with approximately 40 women through participant observation and informal conversations. Twelve women contributed to the arts-based scrapbook over two months (February–April 2025). Women’s engagement varied considerably, ranging from brief exchanges lasting a few minutes to extended conversations spanning multiple visits. Insights also emerged indirectly through staff members (n = 5) who shared contextual knowledge. This range of engagement reflects both the fluid nature of attendance at SWWOP and the ethnographic approach, which values diverse forms of interaction rather than standardised participation. We recognised early in our preparatory work that formal interviewing would not be acceptable to this population group; previous research teams working with SWWOP had been unable to recruit any participants. Our focus was to find a pragmatic but rigorous way to engage with a community who face barriers to engaging in formalised research. The scrapbook was placed within a box that contained craft activities and diamanté art materials, which women often used at the centre.

Prior to the start of the research, we worked closely with staff and support workers at the charity to co-design a methodology that felt appropriate for the context. Many women attending experience severe multiple disadvantage, use illicit substances and are at increased risk of coercion. We were keen to provide a research platform to amplify their voices, but we were mindful of maintaining their safety, confidentiality and avoiding harm for participants and researchers.

Inclusion criteria:

Engages with or has engaged with the organisation.

Aged 18+.

Must have sufficient capacity to consent and participate in the study.

Ethical approval was gained from The University of Sheffield Research Ethics Committee on 10 December 2024 (063995).

#### 2.1.1. Informed Consent from Participants

Consent operated differently for the two methodological components. For ethnographic observations and conversations, we employed a process of ongoing verbal consent. At the beginning of the study period, CB introduced herself to anyone attending the centre as both a volunteer and researcher, explaining that her observations and conversations at SWWOP could be included in research exploring healthcare access. Women were informed they could decline to participate or withdraw at any time, and that declining would not affect their access to SWWOP services. For women who engaged with SWWOP later in the study period, the same introduction process was followed. Staff members helped reinforce awareness that research, supporting women to read the information page in the scrapbook if they were illiterate, and answering any questions about the study if CB was not present. This approach recognises consent in ethnographic research as a continuous, relational process rather than a single transactional event.

For the arts-based scrapbook, consent operated at multiple levels [26]. First, verbal consent was obtained when staff members or CB promoted the scrapbook, explaining the research aims and that contributions would be used in the study. Second, a printed study information page was included at the front of the scrapbook, outlining anonymity procedures and the research’s intended use. This dual approach ensured women understood their creative contributions would be part of a university study. Where possible, a core member of staff was present during these introductions to support participant comfort and safety, and to ensure two people—the staff member and CB—were confident that the participant understood the nature of the research.

Many of the women who engage with SWWOP have substance use disorders, particularly opioids and/or methamphetamine. It was necessary to assess capacity for informed consent in relation to potential cognitive impairment. A brief capacity assessment was conducted by CB in consultation with staff members who knew the women well. To assess capacity, we engaged participants in conversation about the information shared, listening for evidence that they could ‘understand, retain, use and weigh up, and communicate’ that information.

Importantly, no women were formally excluded based on capacity assessment. Rather, if a woman appeared significantly impaired during an initial approach, we delayed seeking consent and returned to the conversation when she seemed more able to engage meaningfully. This approach balanced ethical protection with inclusive participation. We did not exclude participants with substance use disorder per se for two main reasons. First, we had no reliable or appropriate way of determining who was under the influence at any given time without invasive testing that would be stigmatising and misaligned with SWWOP’s values. Second, substance use is a common and often daily reality for many women in this setting. To exclude them would ignore a central aspect of their lived experience and further marginalise their voices.

The decision to obtain verbal, rather than written, consent was intentional. While written consent offers clear documentation and legal protection, many of the women involved in this study have low levels of reading literacy, and we did not want to place unnecessary barriers or pressure on participation. More importantly, verbal consent allowed for a less formal, more personable exchange, which helped to foster a sense of ease and comfort. As a researcher entering a space that was already established as a rare site of safety and support for the women, it was essential that we did not disrupt the existing atmosphere with overly formal processes. Engaging in an open conversation rather than paperwork helped position CB as approachable and respectful of the dynamics of the environment, supporting a more ethical and appropriate process of informed consent. We did not collect any personal demographics, as, in preparatory work, staff suggested this would reduce engagement. There is significant fear associated with ‘institutions’ such as the university, and some women are afraid that they will be monitored or tracked [24]. One risk of verbal consent is the lack of physical documentation, which could make it more difficult to verify that consent was properly obtained if questioned later. However, these risks were outweighed by the benefits, especially given the importance of maintaining the safe, supportive atmosphere already established for the women.

#### 2.1.2. Deliberate Non-Collection of Demographic Data

A key methodological decision was the deliberate non-collection of participant demographic details (age, ethnicity, duration of involvement in sex work, etc.). This decision was made collaboratively with SWWOP staff during study design, based on their extensive knowledge of the women’s needs and concerns. Many women accessing SWWOP have experienced significant harm from institutions—including healthcare, social services, police, and immigration—and associate data collection with surveillance, control, and potential negative consequences. Staff advised that requesting demographic information would likely reduce engagement and could cause distress, replicating the institutional dynamics that contribute to these women’s marginalisation.

This approach aligns with inclusive health research methodologies that prioritise participant safety and willingness to engage over methodological conventions designed for less marginalised populations [24]. Previous research teams working with SWWOP using traditional interview methods were unable to recruit any participants, suggesting that conventional approaches are inappropriate for this context. Our methodology was designed to be pragmatic and responsive to the realities of conducting research with populations who are systematically excluded from traditional research paradigms.

Rather than viewing this as a limitation, we position it as an ethical and methodological strength: the data we collected would not exist had we imposed conventional demographic collection requirements. The richness and authenticity of participants’ accounts—shared in an environment of trust and safety—provides insights that would have been inaccessible through more formalised approaches.

### 2.2. Study Design and Data Collection

#### Ethnographic Approach

This study employed participant observation, a qualitative research method that involves the researcher immersing themselves in the social setting while simultaneously observing and engaging with participants [27]. CB’s role fluctuated along the participant–observer continuum. At times, she engaged in ‘observant participation’—fully participating as a volunteer while remaining attentive to interactions and dynamics. At other times, she adopted a more deliberate observational stance when specific interactions warranted closer analytical attention [28]. This flexible positioning enabled both deep engagement with the community and the analytical distance necessary for systematic observation.

Ethnographic data were collected over a six-month period (October 2024–April 2025) through participant observation conducted once or twice weekly at SWWOP’s drop-in centre and occasionally at the outreach van. This included observations of and conversations with both SSWs and staff members. Staff members were included because they could offer a broader organisational understanding that complemented individual SSWs’ perspectives and provided context for patterns observed across multiple participants.

CB’s position as an existing volunteer at SWWOP prior to the research period facilitated access and trust-building. Some women were already familiar with her and aware of her position as a medical student, while others met her for the first time during the research period. This pre-existing relationship required careful negotiation and transparent communication of the dual roles as both volunteer and researcher throughout the study.

Data were documented through field notes, which included informal writing and voice notes made during or immediately after each period of fieldwork. Field notes served as the primary tool for capturing observations, interactions, and analytical insights. CB recorded detailed descriptions of the physical environment, interactions observed, conversations held (with permission), and her own reflections. These notes combined descriptive content (what happened) with reflexive commentary (CB’s interpretations, questions, and emotional responses).

Additionally, a list of guiding questions was prepared before starting data collection. These questions, derived from the research aims and existing literature on SSW health access, helped structure observations and focus attention on key domains of healthcare access. During analysis, field notes were systematically reviewed, with key passages identified and coded according to the candidacy framework stages (see Section 2.3). The combination of descriptive and reflective field notes allowed for triangulation between what was observed, what participants explicitly shared, and how CB interpreted these data within the broader context of NHS healthcare access.

We used a scrapbook and craft materials to help engage women in discussion as per Dickson et al. [29]. The scrapbook data collection occurred over a two-month period (28 February 2025–30 April 2025). The scrapbook was positioned on the main table in the drop-in centre.

The scrapbook was divided into:

1. Open questions exploring attitudes to and experiences of healthcare
2. Categories of healthcare need for specific feedback

Participants were invited to either write or draw in response to the prompts in the scrapbook.

### 2.3. Rapport, Language, and Data Processing

#### 2.3.1. Rapport-Building

Rapport was established through CB’s sustained presence at SWWOP over the six-month fieldwork period. Trust-building was gradual and relational rather than transactional, developed through consistent attendance, participation in daily activities alongside women (such as preparing food parcels, making tea, and informal conversation), and demonstration of genuine interest in their wellbeing beyond the research aims. CB’s pre-existing volunteer role at SWWOP provided a foundation of familiarity, though building research-specific trust required ongoing negotiation and transparency about her dual role as both volunteer and researcher.

Key strategies for rapport-building included: maintaining informal presence without immediately pursuing research conversations; offering practical assistance; respecting women’s boundaries regarding what they wished to share; and demonstrating consistency through regular attendance, even when research-relevant interactions did not occur. Staff members played an important role in facilitating trust, vouching for CB’s presence and reassuring women about the nature of the research.

#### 2.3.2. Language and Communication

All fieldwork was conducted in English, the primary language used at SWWOP. No translation was required. CB attended closely to participants’ own terminology and phrasing, incorporating their language into field notes and analysis to preserve authentic voice. Colloquial expressions and vernacular were retained in transcription to maintain the linguistic character of participants’ accounts.

#### 2.3.3. Transcription and Data Recording

Field notes were written by CB during or immediately following each period of observation. These combined descriptive content (what happened, who was present, what was said) with reflexive commentary (CB’s interpretations, emotional responses, and emerging analytical thoughts). Voice notes recorded immediately after fieldwork sessions were transcribed verbatim by CB within 48 h to ensure accuracy and maintain immersion in the data. Scrapbook contributions were photographed and transcribed, with visual elements described in accompanying notes. All transcription was conducted by CB, ensuring consistency and deep familiarity with the data.

### 2.4. Analysis

#### 2.4.1. Analytical Approach

Data analysis employed Braun and Clarke’s reflexive thematic analysis (RTA), following their six-phase approach [30,31]. RTA was selected for its epistemological flexibility and alignment with an ethnographic approach, which recognises meaning as contextually produced through the researcher’s interpretive engagement with data rather than discovered as pre-existing patterns [30]. Dixon-Woods et al.’s [21,22] candidacy framework was used as an organising lens within this analytical approach, providing a conceptual structure for understanding healthcare access as a dynamic, negotiated process.

The candidacy framework was selected for its conceptual fit with the study aims and its demonstrated utility in analysing healthcare access among marginalised populations [22,32]. Framework analysis applies an existing theoretical structure while remaining attentive to how data may challenge or extend that structure [21]. This approach was particularly appropriate given the study’s focus on understanding healthcare access as a processual journey, allowing us to examine how SSWs’ experiences mapped onto (and sometimes diverged from) established patterns of healthcare access documented in other marginalised groups.

Data were triangulated across multiple sources (written scrapbook contributions, visual scrapbook contributions, ethnographic field notes, and documented responses to guiding questions) to ensure comprehensive interpretation of findings. Two co-authors (RM and JR) independently reviewed the coded data and its organisation within the candidacy framework. The team then met to discuss the coding, with particular attention to areas of disagreement or ambiguity. Through iterative discussion, consensus was reached on the interpretation and placement of data within the framework stages. This collaborative review process strengthened the credibility and trustworthiness of the analysis.

#### 2.4.2. Data Adequacy

Data collection continued until adequate depth and breadth of understanding were achieved across all seven stages of the candidacy framework. This was evident by the final month of data collection, when ethnographic observations and scrapbook contributions increasingly echoed patterns and experiences already documented, with few genuinely novel insights emerging. The decision to conclude data collection was made collaboratively between CB and the wider research team based on this assessment of data adequacy within the framework. In terms of data saturation, in an ideal research setting, longer observation may have been useful to fully appreciate the vastly complex and heterogenous challenges [33].

### 2.5. Reflexivity and Positionality

CB’s role was shaped by multiple intersecting identities as a woman, medical student, volunteer at SWWOP, and an outsider to the lived experience of street sex work. These positions influenced both how participants perceived her and how she interpreted interactions and data.

As someone without lived experience of street sex work, CB acknowledged she would never fully comprehend the complexity of participants’ lives. Her medical education provided valuable insights into potential areas for NHS service improvement but also aligned her with a healthcare system many participants viewed with suspicion or distrust. Building trust required patience and transparency about the research intentions, and her dual role as volunteer and researcher. CB’s position fluctuated between ‘partial insider’ as a trusted volunteer and ‘outsider’ as an academic researcher and this may have influenced what was shared with her and how.

The co-authors who reviewed the analysis (RM and JR) bring their own positionalities as NHS general practitioners and academic researchers in primary care. Their clinical experience provided valuable context for interpreting healthcare access barriers described by participants. However, this professional positioning also risked bias toward system-level explanations or assumptions shaped by provider perspectives. To mitigate this risk, analysis discussions deliberately centred participants’ own accounts and examined how professional assumptions might be shaping interpretations of the data. Regular reflexive discussions within the team and with SWWOP staff helped surface and interrogate these potential biases throughout the analytical process.

## 3. Results

The candidacy framework is used as an analytical lens to structure the data and explore how SSWs access and experience healthcare; findings from both the ethnographic approach and the arts-based scrapbook are presented together. Whilst the results will be presented in the order of these stages, an SSWs journey through healthcare is rarely linear. Instead, their experiences often involve movement and hesitancy between stages as they navigate complex personal, social, and structural circumstances. The framework is therefore used not to impose a rigid pathway, but to illuminate the experiences through which candidacy is negotiated and realised.

### 3.1. Identification of Candidacy

Identification of candidacy refers to the recognition and interpretation of health symptoms and the decision to seek professional intervention [22]. As the first stage of candidacy, identification is fundamental in receiving adequate care.

For many participants, fear played a significant role in delaying this process. SSWs expressed anxiety about what seeking care might reveal, with one commenting ‘ignorance is bliss’. Some had lived with symptoms for so long they could no longer distinguish what ‘healthy’ felt like and therefore could not recognise abnormalities. Participants described feeling as though once they acknowledged one aspect of their health, it would open the door to a series of other health complications that they had been unaware of. Or, they worried they might discover that health concerns may be more serious than they had realised. Two women, both long-term attendees at the drop-in who CB had come to know over several months, described themselves as ‘hypochondriacs’, explaining that they felt they had to totally avoid thinking about health or they would not be able to stop. Another example of fear was that of test results. Participants described wanting routine STI testing but being too scared of potential diagnoses.

Avoidance of health leads to minimisation of health needs. Many women described having to ‘just get on with it’ and ‘[push] through’. Generally, they stated having to be extremely worried about a health issue to consider seeking healthcare. During a conversation at the drop-in centre, a woman who had been attending SWWOP regularly explained the daily reality of prioritising survival over health: ‘as working women, we don’t get sick pay…we just have to keep working. One woman, visibly unwell with flu during a drop-in session, refused rest despite staff encouragement. CB observed her insisting she needed to collect a food parcel to eat that day, illustrating how immediate survival needs consistently override health concerns.

Minimisation of health needs also extended to delayed presentation to healthcare services. One woman only accessed care at a life-threatening stage, resulting in hospitalisation and coma. Staff at the organisation described a conflicting sense of relief when she was referred to a young person’s care home—they believed this outcome was the only one that would keep her alive. This extreme example illustrates how minimisation can continue until health deterioration becomes critical.

Identification of candidacy involves both identifying a health need and identifying a health service to meet the health need. However, once the women had identified a health need, this often did not lead to seeking professional intervention. Many SSWs preferred to self-diagnose, rather than see an HCP. Internet searches were deemed to be quicker and easier, with the advantage of not having to talk to HCPs. Following on from self-diagnoses, they often opted to self-treat, ignore symptoms or seek advice from other SSW before attending a HCP. Peer advice was particularly sought for substance misuse issues and contraception. One SSW was advised by her GP that the coil would be the most appropriate contraception based on her needs, but she opted for the implant after receiving advice from another SSW.

Interestingly, women were often able to identify the severity of another woman’s health needs before their own. They advised each other to seek medical care or advocated for each other to staff. If a woman had not engaged with SWWOP in a long time, other women informed staff that she was unwell and needed healthcare. This peer advocacy contrasts sharply with the minimisation women applied to their own health needs, suggesting that structural and psychological barriers to identification operate differently when assessing others’ candidacy compared to one’s own.

### 3.2. Navigation of Services

Navigation of services focuses on the idea that a patient has to work to use a service by (a) being aware of the services on offer and (b) having the resources to use the service [22].

Participants described difficulty identifying how to register with a service. Registration systems were cited as confusing and inconsistent, frequently changing formats between in-person, online, or mobile sign-ups. One specific barrier was the lack of a next-of-kin contact, which some GP registration forms required. While organisational staff had previously been able to fulfil this role, changes in policy meant this was no longer accepted, creating an additional obstacle for women without family contacts. Many participants reported difficulties contacting healthcare services due to lack of consistent access to technology. When a phone is provided by an organisation, it is often stolen or sold. This makes appointment reminders and other clinical communication impossible to receive. Staff at SWWOP attempted to mitigate this by reminding women about appointments, including asking other women to pass on reminders. However, this informal system was insufficient to compensate for the lack of consistent, direct communication channels.

*In the scrapbook, one woman wrote… ‘I need a clear way to contact them’*

To travel to an appointment, women need to know where it is and be able to get there. Without constant access to the internet, it can be difficult for the women to work out how to get there. Additionally, the cost of public transport is often too high. The organisation tries to provide bus passes to help with this.

### 3.3. Permeability of Services

Permeability of services is concerned with the ease with which people can use services [34]. Barriers to permeability can be administrative, or due to misalignment between a service and its user—essentially whether or not the patient feels comfortable using the service [34].

Participants described feeling uncomfortable when receptionists asked personal questions about their health, feeling that this violates their privacy. This has become a common policy in NHS general practice, with a reason for the appointment needed in order to access an appointment.

*In the scrapbook, one woman wrote…; ‘Not having to tell the receptionist personal issues’ ‘receptionist asking personal questions not good’*

The time it takes to register for some healthcare services was described as an problem. One participant was told by an NHS GP service that registration would take two weeks; she declined, believing she might no longer need care by then. In contrast, when offered immediate registration with SWWOP’s internal GP, she complied readily despite having no health concerns at that time.

Participants reported administrative constraints to accessing appointments. During a group conversation at the drop-in centre, one woman recounted her frustration at walking for 40 min to her GP practice to try to make an appointment, only to be told on arrival that they only accepted telephone requests. Many women have no access to a smart phone, so digital access is impossible. Even when women had phones, they often preferred face-to-face appointments over telephone appointments due to the cost of phone calls. One participant explained that phone appointment costs caused her to rush through consultations to keep bills lower, preventing her from fully discussing health concerns.

*In the scrapbook, one woman wrote…; ‘Go in person not just on phone’*

Many participants experienced being barred from GP services. Reasons for this include missing previous appointments and displaying triggered behaviours. This prevents the women from accessing further appointments and deters them from registering with alternate services.

### 3.4. Appearing and Asserting Candidacy

Appearing and asserting candidacy is the act of being able to formulate, articulate and seek help for an issue [34]. This may be harder for vulnerable groups due to power imbalances, making it more difficult for them to ask for help.

Minimisation of need was a recurring theme. Many participants acknowledged that they felt their health was unimportant, which made it harder for them to assert a claim. Repeated experiences of social exclusion and trauma eroded their self-worth and confidence to seek help. In discussing reception practices, a woman who had been engaging with SWWOP services for several years expressed a view shared by many: *‘We already find it hard to tell the doctor personal things, we don’t want to be telling everyone.’* She described feeling exposed when asked to explain her health concerns in front of other patients. They feared being heard by other patients in the waiting room, especially men. Women often reported feeling particularly vulnerable in the presence of men when seeking healthcare. Male HCPs can be an even greater trigger, due to traumatic past experiences with men, particularly those deemed to be in positions of authority. This compounds feelings of unsafety, making SSWs less likely to seek care.

It was clear that positive experiences with empathetic healthcare providers were transformative. This played a crucial role in the process of appearing and asserting themselves to HCPs in the future. The importance of having female HCPs was echoed here.

The participant’s gratitude for SWWOP’s internal GP played a large role in their confidence to assert. The understanding that she was there for them, and had chosen to treat them because she specifically wanted to help SSWs allowed them to feel that they deserved treatment. One participant added “[love] yourself, we deserve it”, verbally explaining further that believing that they were worthy of love was the first step to asserting their needs.

*In the scrapbook, one woman wrote…; ‘when I hear about healthcare I think caring People. Nice people. People who want to help. I feel it’s people to help care for unwell people.’*

In response to the prompt ‘What would make you trust your doctor more?’, one participant drew a heart, verbally stating she just wanted to be treated with kindness.

The main consensus here was that the participants wanted to feel respected and trust a HCP enough to assert a claim. The value of friendly, consistent HCPs who showed they were really listening to the women was paramount to their ability to assert their claim to candidacy.

### 3.5. Adjudication by Healthcare Professionals

Adjudication is the influence that a HCPs judgements and decisions have on access and experience [34].

This is particularly important for SSWs as they described experiencing judgemental attitudes from HCPs often. Participants discussed feeling as though they were not deemed as deserving of care due to their identity as SSWs. Some argued that they felt as though they were viewed as making bad decisions and were therefore incapable of making decisions about their own health. The issue of being asked personal questions by receptionists is also relevant here. This led SSWs to feel as though they were being stigmatised and pigeonholed before they saw an HCP. The feeling of alienation also occurred from being barred from services. This demonstrates a decision from professionals surrounding the validity of a SSWs claim to candidacy. This adjudication was particularly evident in mental health services, where participants reported it was ‘hard to get nurses to come out,’ suggesting that professionals’ assessments of risk or need determined service access rather than participants’ own expressions of candidacy.

Participants described having to fight for healthcare, whilst HCPs ultimately decide on the outcome. In response to the prompt, ‘What do you think about when you hear the word healthcare?’, one participant wrote, ‘That’s all I’ve heard for 15 years.’ This response reflected a sense of persistent engagement with healthcare services, often initiated by professionals rather than the individual. Several participants described experiences where healthcare interventions, particularly types of contraception, were pursued or offered repeatedly, despite their own uncertainty or reluctance. In these instances, decisions about care appeared to be made primarily by professionals, with women feeling that their preferences were secondary or disregarded. These accounts suggest that adjudication did not always follow a claim of candidacy from the individual; instead, candidacy was sometimes externally imposed, with professionals determining the need for care irrespective of the participant’s own assessment.

### 3.6. Offers of/Resistance to Healthcare Services

Patients can resist offers of healthcare, such as referrals or medication, directly affecting access [34]. Among SSWs, resistance was often shaped by prior negative experiences. This includes refusing healthcare interactions with male HCPs due to fear. This was particularly relevant for intimate health concerns, such as sexual health or mental health issues.

Resistance to substance misuse treatment was common. This was largely due to fear of bad side effects, particularly withdrawal. Many of the women had previously engaged with substance misuse programmes, using methadone tablets to treat opioid dependency. They explained that after having negative experiences with this treatment, when offered buprenorphine injections (an alternative treatment), they were hesitant and declined. However, once positive experiences with buprenorphine injections were shared within the community, more women opted in. There were many reasons for this. Some participants did not want to commit to remembering to either collect prescriptions or take medication. Others felt that they had a difficult relationship with drugs and did not trust themselves to use them safely. Others worried about side effects. Mental health medication was frequently declined.

There were examples of resistance to antenatal and perinatal care. A large reason for this was due to fear of child removal by social services. One woman explained that she refused skin-to-skin contact after birthing her child, as she did not want to develop a greater emotional attachment to a child that would shortly be removed from her care.

### 3.7. Operating Conditions and Local Production of Candidacy

The operating conditions and local production of candidacy stage refers to the ‘perceived or actual availability and suitability of resources to address […] candidacy’ [34]. This includes the local influence on patient and HCP relationships.

A clear theme from this study is the time it takes to get an appointment, particularly for mental health services. All participants stressed the importance of getting an appointment or assessment quickly. Long waiting lists for mental health services can feel like a total rejection from the service, leaving patients feeling alone. This prevents them from seeking further care. Long wait times between seeking an appointment and the appointment itself increased the likelihood of navigation and permeability barriers occurring—such as forgetting appointments due to a lack of phone reminders. This then increased the risk of being barred from a service, creating a cycle in which women no longer sought appointments. Operating conditions, therefore, did not merely create isolated barriers, but generated cascading consequences across multiple stages of the candidacy framework.

*In the scrapbook, one woman wrote…; ‘assessments are slow, getting help us slow, needs to be face to face’ and ‘getting appointments quickly no phone wait’.*

Time constraints during appointments are also a barrier. SSWs are more likely to require more time and space to assert candidacy, due to trauma and fear of judgement. They are likely to have prior negative experiences, which mean that building trust with an HCP takes longer. Being rushed through an appointment can make them feel dismissed, making them feel unworthy of care.

*In the scrapbook, one woman wrote…; ‘feeling that you are not be rushed’*

Lack of consistency is a barrier. This applies to inconsistent appointment booking systems. Navigating a new system can be more difficult for SSWs, so changing systems make it less likely for them to seek care. Continuity with a HCP is also important, as it allows SSWs to build rapport and trust with a HCP.

An example of this is SWWOP’s internal GP. The consistent time and care that she has offered the woman allows them to feel safe and trust her. Often, due to barriers discussed, SSWs miss their appointments with her. This means it is harder for the organisation to prove her necessity, as uptake is low. However, utilisation is not an appropriate measure of need, and in this case, the barriers that cause low uptake are the reasons why the internal GP is so essential. Paradoxically, this risks decreased funding for the GP service, as utilisation metrics fail to account for the structural barriers that prevent attendance. This illustrates how operating conditions can create policy decisions that worsen access for those who most need services.

#### Application of the Candidacy Framework

The candidacy framework provided a useful organising structure for understanding SSWs’ healthcare access experiences, though the strength of evidence varied across the seven stages. Data strongly supported all seven stages, with particularly rich findings emerging around identification of candidacy (where fear and minimisation were dominant themes), permeability of services (where administrative barriers were extensively documented), and appearing and asserting candidacy (where the role of empathetic, trusted providers was central).

Some framework stages were less richly represented in the data. For example, while adjudication by healthcare professionals was evident in participants’ accounts, most data focused on negative adjudication experiences; positive adjudication examples were largely limited to SWWOP’s internal GP. Similarly, offers of/resistance to healthcare had clear examples but fewer data points compared to earlier framework stages, possibly reflecting that many participants struggled to reach this stage of the healthcare journey.

Importantly, some participant experiences challenged the framework’s conceptualisation of healthcare access as a linear journey. SSWs’ experiences were often cyclical—moving forward and backward between stages, or experiencing multiple stages simultaneously. For instance, women might successfully navigate to an appointment but then fail to appear due to renewed fear or minimisation of need. The framework also does not explicitly capture the role of peer networks in shaping candidacy; SSWs’ trust in other SSWs’ healthcare recommendations over HCPs’ advice represents a pattern of alternative candidacy construction not addressed in Dixon-Woods et al.’s original formulation [34].

Additionally, the data revealed how operating conditions created cascading effects across earlier stages—for example, long wait times leading to forgotten appointments, resulting in being barred from services, which then deterred future registration attempts. This interconnectedness suggests the framework’s stages are even more deeply interrelated than the original conceptualisation acknowledges. The impact of intersectionality and layers of disadvantage for these women was apparent and potentially under-represented in the candidacy framework [35].

Despite these observations, the framework successfully illuminated the processual, negotiated nature of healthcare access for this population and provided a coherent structure for analysing diverse data sources.

## 4. Discussion

There are significant barriers to access to healthcare for SSWs. Participants face personal barriers, such as fear of one’s own health and hypochondria, and structural obstacles, such as consistent telephone access and time constraints.

### 4.1. SSWs Are Systematically Excluded, Not Disengaged

While barriers were widely reported, participants often placed culpability on systems rather than individual HCPs. The findings show that services are not structured towards the needs of SSWs, consistent with the existing literature documenting systemic barriers to healthcare for sex workers [8,36]. In contrast to some literature that portrays a uniformly negative relationship with healthcare [9], many respondents often expressed genuine gratitude for the positive experiences they had—particularly in settings where care was consistent, trauma-informed, and non-judgemental [37]. This suggests that SSWs are not wholly disillusioned with healthcare but rather frustrated with inconsistencies in systems and practice.

This optimism may reflect the specific context of their relationship with the organisation’s GP, a rare example of continuity, safety, and trust; but it challenges narratives of disengagement [9] and shows that meaningful, sustainable access is possible if systems are flexible enough to meet people where they are.

### 4.2. Healthcare Access for SSWs Is a Process, Not a Singular Event

The process of accessing and experiencing healthcare for SSWs fits into the candidacy framework [34]. This framework provides a clear structure for analysing healthcare access not as a singular event but as an evolving process [22,32]. Traditional models often rely on a utilisation-based approach—measuring access by counting service use or attendance—which can overlook the many barriers, refusals, and informal attempts that occur before or instead of formal engagement [38,39]. Using this approach has enabled a more nuanced analysis of healthcare experiences. This processual perspective reveals that the challenges SSWs face are not simply about their willingness or ability to use services but are embedded in systemic structures and attitudes that limit genuine access [22,34].

### 4.3. SSW Solutions

A notable aspect of this research is its attention to participant voice. Many studies have focused solely on documenting barriers; here, participants also suggested solutions. SSWs are under-reached, and therefore, many previous studies have consulted either healthcare providers or charity staff rather than sex workers themselves [4,36]. This study has captured raw perspectives from SSWs themselves [7], allowing for the development of solutions that SSWs are comfortable with. This underlines the importance of moving away from top-down healthcare design and towards models that are co-produced with service users [24].

### 4.4. What SSWs Want in a HCP

The SSWs in this study are clear about what is important to them in a HCP. Many SSWs expressed difficult experiences with male HCPs and preferences for female HCPs [40]. The importance of openness and friendliness in HCPs was expressed. SSWs are consistently judged and stigmatised in every area of life [41,42]. Kindness from HCPs creates a uniquely safe environment where SSWs can be offered equal respect and care.

SSWs want to be heard and listened to [43]. This demonstrates an understanding from the HCP that SSWs are capable and worthy of making decisions about their lives and health. HCPs who actively listen amplify the voices of SSWs, who are often unheard. This can create a special sense of empowerment in a usually disempowered situation [44].

### 4.5. Digital Healthcare Does Not Work for Everyone

As healthcare becomes increasingly digital, the risk of excluding SSWs from healthcare grows. Whilst the digitalisation of healthcare has the potential to increase accessibility for certain patient groups, it is important for policy makers and HCPs alike to be aware of the potential inequalities it can introduce. Greenhalgh suggests that patients who are disadvantaged by digital exclusion could receive a flag on their health record, reminding HCPs and staff to offer less digital or non-digital options [45]. Expanding on Greenhalgh’s work on digital exclusion, SSWs could be offered healthcare services that consider their fast-changing access to mobile phones [46].

### 4.6. Comparison with Existing Literature

The health needs and barriers to healthcare identified in this research are in line with previous studies investigating health needs for SSWs [3,4,7,9,19]. This research has expanded on how these barriers emerge and persist by examining the interplay between SSWs and services [9].

Much of the research directly engaging with SSWs was conducted prior to 2016 [4,5,7,36], with more recent studies tending to review the literature [47] or interview professionals working around SSWs [3]. This study is distinct in that it engages HCPs, charity staff and SSWs, but crucially re-centres the voices of SSWs themselves. The NHS has changed considerably in the last decade, with particular focus on digitalisation [45]. As such, the study offers a timely and original contribution by foregrounding the lived experiences of SSWs navigating a rapidly evolving NHS.

As with this study, Mastrocola et al. carried out research with a specific third-sector organisation [7]. Both Mastrocola et al. [7] and Jeal et al. [34] noted the advantage of harnessing the role of the voluntary sector in SSWs’ health. This study reinforces these findings by demonstrating how the NHS can work with third-sector organisations to maximise ease and efficiency for SSWs.

Unlike other studies that concluded that SSWs are disengaged from healthcare [5], this study found that many participants were engaged but excluded. This reframes the issue not as one of individual disengagement but of systemic exclusion within the current healthcare landscape.

Mastrocola et al. [7] introduced the concept of candidacy to understand their results, particularly focusing on the permeability stage. This study expands on this idea by mapping and interpreting results within the candidacy framework. Being the first study to comprehensively apply the candidacy framework to SSWs is significant because it opens new analytical ground and offers a novel perspective on how access issues are experienced and addressed within this marginalised population. This original application also provides a foundation for future research, allowing others to build on, adapt, or challenge the candidacy framework in relation to healthcare access for SSWs.

### 4.7. Strengths and Limitations

#### 4.7.1. Strengths

This study was developed with an inclusive health research methodology that was participant- and community-focused/led [24]. We employed a flexible approach to data collection (people could add to the scrapbook at any time), and data could be collected in a familiar, trusted environment with a familiar face. This project has helped to build links with a population who are often excluded by the design of research methodologies, and we hope we can build future interventions driven by those in most need.

#### 4.7.2. Limitations

While this study offers valuable insights into healthcare access among sex workers (SSWs), several limitations should be acknowledged. The majority of participants were cis-female, and the results may not be applicable to transgender or cis-male SSWs, and the study’s limited time frame constrained opportunities to build deeper rapport with participants. We deliberately chose not to collect participant demographic details. While this limits our ability to present traditional demographic profiles of participants, this decision was essential to enabling participation. The women who engage with SWWOP have well-founded fears of institutional data collection, and requiring such information would have replicated harmful dynamics and likely prevented engagement entirely. This methodological choice reflects the pragmatic realities of conducting inclusive health services research with highly marginalised populations, and aligns with calls for research methodologies that prioritise participant safety over convention [24]. The absence of demographic identifiers does not diminish the validity or transferability of the experiential accounts provided; rather, it enabled access to perspectives that would otherwise have remained unheard.

Reliance on a single service for recruitment may have narrowed the sample, as different cities have different service delivery models and organisational structures, and the research was embedded in a specific organisational environment that may not be replicable in other service settings. The scrapbook method, while receiving contributions, ultimately served as a conversation starter rather than producing visual results, indicating that the methodology of this study could be better edited to align with SSWs’ needs, and participants were more responsive to general healthcare prompts than specific sexual health ones.

The arts-based scrapbook was intended to offer a flexible and participant-led mode of expression [29]. However, it was noted that participants preferred to write in the book or verbally respond to prompts. This likely reflects a combination of factors: unfamiliarity with the method, limited trust and time constraints. This outcome provides insights into the limits of creative methodologies. While creative methods like this are often recommended for working with marginalised groups, the low engagement observed here suggests that the effectiveness of such methodologies can be dependent on factors such as study design, environment, support provided and level of trust between researcher and participant.

Building rapport with SSWs is a time-intensive process that should not be underestimated—this is in line with Dickson’s findings from her ABR with ‘vulnerable’ women [29]. Without a solid foundation of trust, creative or participatory methods may inadvertently increase discomfort or reluctance to engage, rather than facilitate it. In this study, it appears that some participants may have felt uncertain about using the scrapbook method before a trusting relationship was established.

### 4.8. Implications for Research

This study suggests several avenues for future research to broaden the scope of sex workers (SWs) by including transgender SWs, male SWs, and those engaging in non-traditional sex work types to compare results to the wider SW population. Healthcare research should explore specific areas like long-term physical health, mental health, substance use, sexual and reproductive health, and emergency care, while accounting for differences in financial access to these services. Intervention design research should work with SSWs to design and evaluate better services.

### 4.9. Implications for Practice

Based on insights from this study, several recommendations can be made to improve NHS healthcare access and experience for sex workers (SWs), including designing health systems that avoid excluding groups before they reach services and shifting towards theoretical models that include interpersonal and systemic indicators of unmet need. SWs should be understood as disadvantaged in healthcare access rather than disengaged, with appointments made more readily available and emphasis on ‘walk-in’ options rather than being barred for exhibiting triggered behaviours [47]. SSW-specific GPs should be considered to build trust through consistent care. Creating safe spaces for SWs to access healthcare is important, as mainstream care settings can be triggering. There should be caution around increasing digitalisation of healthcare, with no-digital or less-digital options provided for SWs who may face barriers, and appointment booking systems should allow for looser time constraints and more flexibility. Healthcare providers need proper training and understanding of triggers that affect SWs in medical settings.

Medical training should include education on trauma-informed care, with specific attention to the needs of populations facing multiple exclusions. Developing a curriculum that trains professionals to recognise and dismantle access barriers could result in more equitable healthcare provision.

Empowerment through rights-based education programmes for SSWs also offers a valuable means of improving the problem of exclusion from services and increasing access to care. Community-based approaches that are tailored to the specific community are essential. For this to be achieved, a holistic, multi-disciplinary approach is required to help break cycles of addiction and mental health, avoiding silos of fragmented, speciality-led care.

## 5. Conclusions

This study aimed to build on existing research demonstrating unmet health needs for SSWs. SSWs are a highly marginalised group who often experience severe multiple disadvantage. This causes SSWs to be at risk of vast health inequalities. The health needs of SSWs are interlinked and interdependent, meaning they cannot be solved on a case-by-case basis, but rather need a systemic overhaul. The barriers and enablers of access to adequate NHS healthcare provision for SSWs have been explored using the candidacy framework. These findings demonstrate the advantage of conceptualising NHS healthcare access and experience for SSWs through a model of candidacy. The importance of trauma-informed, consistent care should not be overlooked. Future research should explore alternative care models for SSWs and should be co-produced with SSWs themselves.

## Figures and Tables

**Table 1:** Summary of relevant sex work types, adapted from Hester et al. [1].

Category	Definition
Parlour-based work, brothel work	Premises explicitly for providing sex, often more security than on street.
Escort	Service pre-planned and provided for specific clients indoors.
Online sex work	Sex work organised and provided online. Includes pornography and webcamming.
Street and outdoor	Solicited or serviced on streets or other public areas.

**Table 2:** Summary of the stages of the candidacy framework, adapted from Dixon-Woods et al. [21,22].

Stage of Candidacy Framework	Dixon-Woods	In Practice
Identification of candidacy	How people recognise their symptoms and respond to them.	Do these symptoms require medical attention?
Navigation of services	Awareness of available services Ability to mobilise a range of practical resources	Which service do I need? How do I find it? How do I get to it?
Permeability of services	The ease with which people can use services More porous—less qualifications, less mobilisation of resources Less porous—more qualifications, more mobilisation of resources	Am I eligible for an appointment? What needs to be done for me to book an appointment?
Appearing and asserting candidacy	A person making a claim to candidacy for medical attention or intervention	Do I know what my needs are? How do I articulate and explain these?
Adjudication by healthcare professionals	The judgements and decisions made by healthcare professionals which influence a person’s progression through healthcare	What do healthcare professionals think of my claim? Do healthcare professionals think I deserve medical attention/intervention?
Offers of/resistance to healthcare services	The choice of a person to refuse a service they have been offered	Do I want to see this healthcare professional? Do I want this treatment? Do I feel judged by this offer?
Operating conditions and local production of candidacy	The influence of local services, resources and availability	Does this service have what I need? Do I feel able to present myself to this service?

## Data Availability

No new data were created or analyzed in this study. Data sharing is not applicable to this article.

## Abbreviations

A&E	Accident and Emergency
ABM	Arts-Based Method(s)
ABR	Arts-Based Research
DERA	Deep End Research Alliance
GP	General Practitioner
HCP	Healthcare Professional
HIV	Human Immunodeficiency Virus
NHS	National Health Service
PRISMA	Preferred Reporting Items for Systematic Reviews and Meta-Analyses
STI	Sexually Transmitted Infection
SW	Sex Work/Sex Worker
SWs	Sex Workers
SSW	Street Sex Work/Street Sex Worker
SSWs	Street Sex Workers
UK	United Kingdom
